# Acute Exposure to a Common Organic UV Filter Does Not Alter the mRNA of Gonadal Estrogen or Growth Hormone Receptors in Mozambique Tilapia (*Oreochromis mossambicus*) In Vitro

**DOI:** 10.3390/genes16111357

**Published:** 2025-11-10

**Authors:** Glenna Maur, Kelly Silva-Picazo, Camila Dores, David Marancik, Euan R. O. Allan

**Affiliations:** 1 Department of Pathobiology, School of Veterinary Medicine, St George’s University, St. George’s P.O. Box 7, Grenada; 2 Department of Microbiology and Immunology, School of Medicine, University of Nevada, Reno, NV 89557, USA; kellysilva@med.unr.edu

**Keywords:** bioaccumulation, endocrine disruptor, estrogen receptor, growth hormone receptor, *Oreochromis mossambicus*, oxybenzone, recreational waters, wastewater processing plant

## Abstract

Background/Objectives: Organic UV filters are chemical compounds that are commonly used in sunscreen products to absorb UV radiation from the Sun. To date, the filters have been detected in aquatic environments worldwide, as well as in aquatic organisms, including fish and coral. Hydroxy-4-methoxybenzophenone (BP-3) is a common organic UV filter and it is well documented to be among the filters that are detectable worldwide in water samples and aquatic organisms. Long-term exposure in vivo studies have demonstrated that high doses of BP-3 can cause endocrine-disrupting effects in aquatic organisms. Methods: Using gonadal cell culture and quantitative RT-PCR, our study aimed to ascertain the effect of environmentally relevant doses of BP-3 (detected in aquatic systems) on the gene expression of reproductive targets, estrogen and growth hormone receptors (ERs and GHRs), in Mozambique tilapia (*Oreochromis mossambicus*) after an acute 24 h treatment. Results/Conclusions: Our study is the first to use an in vitro design to investigate the mechanism of the action of BP-3 on gonadal tissue in fish. Our results show that BP-3 does not induce gene regulation directly on the gonads of tilapia at doses that are comparable to what is detectable in aquatic environments after 24 h. We do verify, as seen in other teleost species, homologous regulation of ERβ in male tilapia gonadal tissue.

## 1. Introduction

Organic ultraviolet (UV) filters are a common ingredient in sunscreens and other personal care products that have gained attention in recent years due to their ubiquitous presence in aquatic environments worldwide [1,2,3,4]. These organic UV filters, also known as chemical filters, are lipophilic molecules that absorb UV rays [4,5,6], which is why they are extensively used as an active ingredient in sunscreens [4,5,7]. They are also commonly found in cosmetic products and plastics to prevent degradation from the Sun [7]. These chemicals enter aquatic ecosystems via wastewater systems from household sewage and factories, and through human recreational activities, such as swimming and surfing [8]. They have been detected in both fresh and marine bodies of water, more frequently in populous areas, but also in remote and less populated regions like the Arctic [8,9,10,11,12,13]. On average, samples have been detected on the scale of ng/L to low µg/L (see Montes-Grajales 2017 [8] for a review of locations and concentrations). Studies examining influent and effluent wastewater removal plant samples demonstrate that some wastewater plants are less than optimally efficient at removing organic UV filter contaminants [14].

Hydroxy-4-methoxybenzophenone (BP-3 or oxybenzone), the most common organic UV filter used in sunscreen for the past forty years [3], has been recently detected in water samples from aquatic systems worldwide [2,7,12,15,16,17]. Detectable levels are typically on the scale of ng/L; however, one study reported concentrations of BP-3 and as high as 1400 µg/L from inshore waters [18]. BP-3 can be persistent in aquatic ecosystems, with its half-life ranging from a few weeks to a few months, depending on water conditions, and shows a decrease in half-life with direct exposure to sunlight [19].

BP-3 is well documented to be one of the organic UV filters that is capable of bioaccumulating within fish tissue [2,15,16,20]. It is a highly lipophilic substance, contributing to its ability to accumulate in adipose tissues of living organisms [21]. It has been detected in fish and prawns at concentrations ranging from 1.46 µg/kg to 151 µg/kg [12,17,22]. Samples analyzed from lionfish muscle tissue from Grenada showed BP-3 to be the most frequently observed organic UV filter detected, consistent with the finding that BP-3 was the most prevalent filter detectable in Grenadian water samples [18]. Organic UV filters may pass from prey to predator and bioaccumulate at higher levels in animals at the top of the food chain, demonstrated by observation of higher concentrations of these filters detected in apex predators, such as the lionfish [17], dolphins [23], and cormorants [12], compared to the concentrations detected in the aquatic systems they inhabit.

There is an emerging body of evidence suggesting that BP-3 has estrogenic endocrine-disrupting effects on a wide taxonomical range of aquatic organisms [24,25,26,27,28]. Immersion in BP-3 can cause an increase in plasma vitellogenin in males [29], a female-biased population [25], a failure to reach gametogenesis in both sexes [25], and a reduction in egg production and hatching [30]. However, estrogenic endocrine-disrupting effects are inconsistent between studies and the doses used in these studies are higher than those that occur in natural environments.

To date, all previous studies examining the potential of BP-3 as an endocrine disruptor in fish have used an in vivo model. In vivo study designs have inherent variables, such as host-specific physiological effects and environmental conditions, that may influence interpretations about the direct mechanisms of action of BP-3. As discussed above, exposure to BP-3 can induce phenotypic changes in fish in vivo that suggest estrogenic effects, but the mechanism of these effects has not been thoroughly examined. Mozambique tilapia (*Oreochromis mossambicus*) have been used as a model for studying endocrine disruption and reproductive endocrinology [31,32,33]. Additionally, tilapia are widely distributed in tropical aquatic systems, including where BP-3 contamination has been reported, making them ecologically relevant and a potential indicator of environmental risk [34,35]. The present study investigated the mechanisms of the action of BP-3 on reproductive targets in testes and ovarian follicles of Mozambique tilapia in vitro to examine the direct effects of BP-3 on gene expression in these tissues while eliminating the variables that arise during in vivo studies. This study focused on examining environmentally relevant concentrations of BP-3 on gene regulation. The mRNA levels of growth hormone receptors (GHRs) were measured because in vivo studies exposing fish to organic UV filters similar to BP-3 have demonstrated a decrease in the body length of larvae [24]. The mRNA levels of estrogen receptor isoforms (ERα and ERβ) were also measured to ascertain their estrogenic effects due to BP-3 exposure. The current study aimed to find a possible pathogenesis of the endocrine-disrupting effects of BP-3 on fish, as previously observed in vivo.

## 2. Materials and Methods

### 2.1. Animals

The protocols and procedures of this study were approved by the St. George’s University Institutional Animal Care and Use Committee (IACUC-19005-R). Fish collection permits were obtained from the Grenada Ministry of Agriculture, Forestry, Lands, and Fisheries. Mozambique tilapia were caught from freshwater rivers and lakes in Grenada, West Indies, and housed at the Aquatic Animal Medicine Research Laboratory at St George’s University for 90 days, in tanks under filtered, recirculating, 27 °C water conditions. Fish were held at 8:14 light/dark cycles and fed a commercial feed. Ten female adult tilapia (102.4 g ± 23.0 standard deviation (SD)) and fifteen male adult tilapia (93.8 g ± 19.4 SD) were used in this study. Fish were euthanized by immersion in >250 mg/L of tricaine methanesulfonate (MS-222), in accordance with SGU’s IACUC (IACUC#19005-R, approved 10 May 2019 by St. George’s University).

### 2.2. Cell Culture

Fish were euthanized and ovarian follicles and testes were bluntly dissected. Two distinct sets of samples based on sex were created (male and female), each with *n* = 5 for all treatment groups. As each cell culture well required 150 g of tissue, it was necessary to pool gonadal tissue into replicate groups. For each female replicate group, ovarian follicles from two fish were pooled. For each male replicate group, testes tissue from three fish were pooled. Gonadal tissue was rinsed twice, each time with 5 mL phosphate-buffered saline (PBS), using a disposable pipette to reduce chances of contamination during the culture process.

Gonadal tissue was finely chopped (~0.5 mm slices) using a razor, and 150 mg of tissue from a given pooled replicate was deposited into a single well, for a total of six wells per replicate in a 24-well tissue culture plate. Leibovitz’s 15 media (L15) was combined with 10% fetal bovine serum (FBS) and 1% penicillin-streptomycin, hereafter referred to as L15 (complete). One mL of L15 (complete) was added to each well and then incubated for 1 h at 25 °C prior to treatment. Wells were visually inspected after the pre-incubation period to ensure no contamination.

The tissue in each well was then treated with BP-3. Each replicate group of tissue underwent six separate treatment groups: group 1 received 1 mL of a 1% solution of ethanol in L15 (complete) as a negative control; groups 2 through 5 were treated with 1 mL of increasing doses of BP-3 in L-15 (complete) (0.1 µg/L, 1 µg/L, 10 µg/L, 100 µg/L); and group 6 was treated with a 1 mL solution of 20 µg/L of estrogen (E2) in L15 (complete). BP-3 and E2 were dissolved using ethanol. After treatment, cells were incubated for another 24 h at 25 °C to allow time for any effect to take place. These culture conditions have been developed based on previously described cell culture methods for Nile tilapia [36,37].

### 2.3. RNA Extraction and qPCR

Using a PureLink™ RNA Mini Kit (Thermo Fisher Scientific, Waltham, MA, USA), the total RNA was extracted from cultured gonadal cells of tilapia and converted to cDNA using iScript™ RT Supermix (Bio-Rad Laboratories, Hercules, CA, USA), as per the manufacturer’s instructions. Total RNA purity and concentration were measured using a Nanodrop spectrophotometer. Quantitative RT-PCR (*q*PCR) was conducted using SsoAdvanced™ Universal SYBR Green Supermix (Bio-Rad Laboratories), using 20 µL of total reaction volume to quantifiably measure the mRNA levels of ERs, GHRs, and β-actin. Primers for the following *Oreochromis mossambicus* mRNA sequences were used: ERα (Forward 5′-GGCTCAGCAGCAGTCAAGAA-3′, Reverse 5′-TGCCTTGAGGTCCTGAACTG-3′); ERβ (Forward 5′-CAGTGCACTATTGACAAGAACCGAC-3′, Reverse 5′-CCAGCATGAGGATCTCCAACCAGC-3′); GHR (Forward 5′-CACACCTCGATCTGGACATATTACA-3′, Reverse 5′-CGGTTGGACAATGTCATTAACAA-3′); and β-actin (Forward 5′-TACCACCATGTACCCTGGCATC-3′, Reverse 5′-TACGCTCAGGTGGAGCAATGA-3′). All primers were validated in-house and previously published [38,39,40]. The thermocycler program used was as follows: 95 °C (2 min), 50 °C (2 min) followed by 40 cycles of 95 °C (10 s) and 60 °C (15 s). The mRNA expression levels for ERs and GHRs have been normalized to β-actin and compared to the control samples.

### 2.4. Data and Statistical Analyses

Statistical analysis was completed using GraphPad Prism software, version 8. A one-way ANOVA was conducted on all results to determine any statistically significant differences in mRNA expression levels (significance level *p* < 0.05). Statistically significant results were followed with Tukey’s multiple comparison test (significance level *p* < 0.05).

## 3. Results

### ERα, ERβ, and GHR Regulation in Tilapia Gonads Following BP-3 Exposure

**Figure 1:**
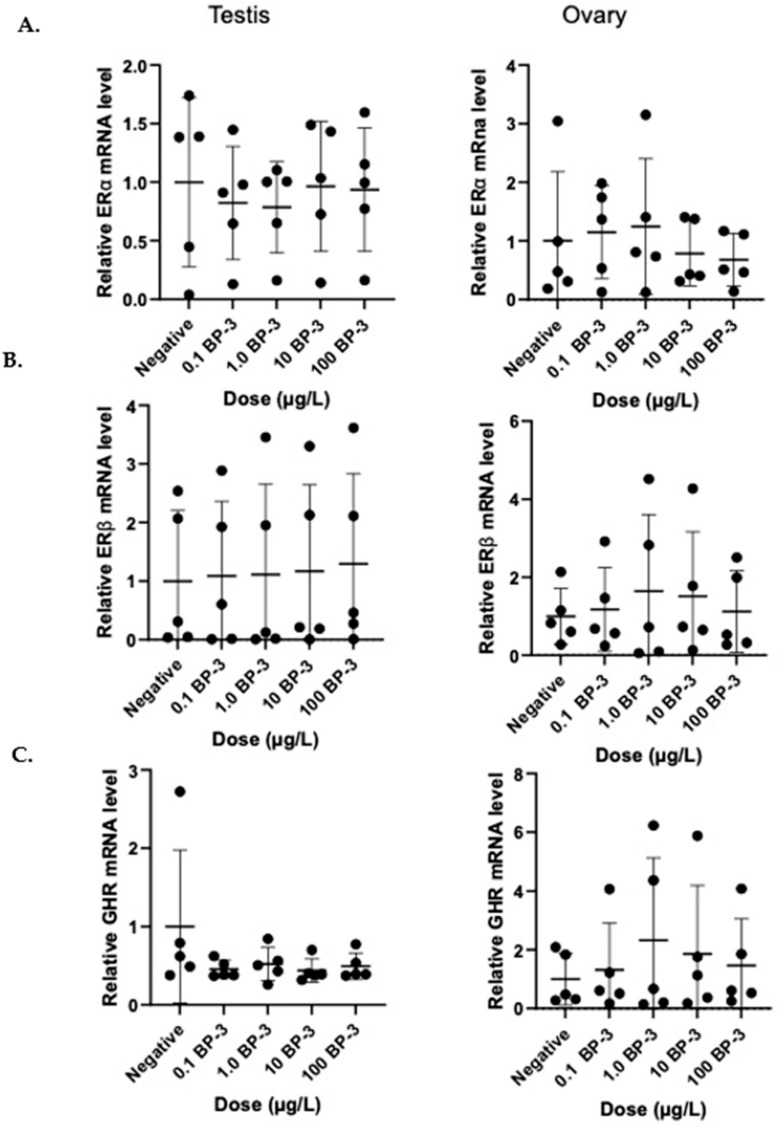
In vitro treatment of BP-3 for 24 h does not alter mRNA gene expression in tilapia (*Oreochromis mossambicus*) gonad tissue of (**A**) ERα, (**B**) ERβ, or (**C**) GHR. Gonadal tissue was cultured and treated with 0 µg/L, 0.1 µg/L, 1.0 µg/L, 10 µg/L, or 100 µg/L BP-3 for 24 h at 25 °C (n = 5 for each treatment group). Expression levels were determined by quantitative real-time PCR (qPCR) and are presented relative to the internal control gene (β-actin), with respect to control (mean ± SD).

**Figure 2:**
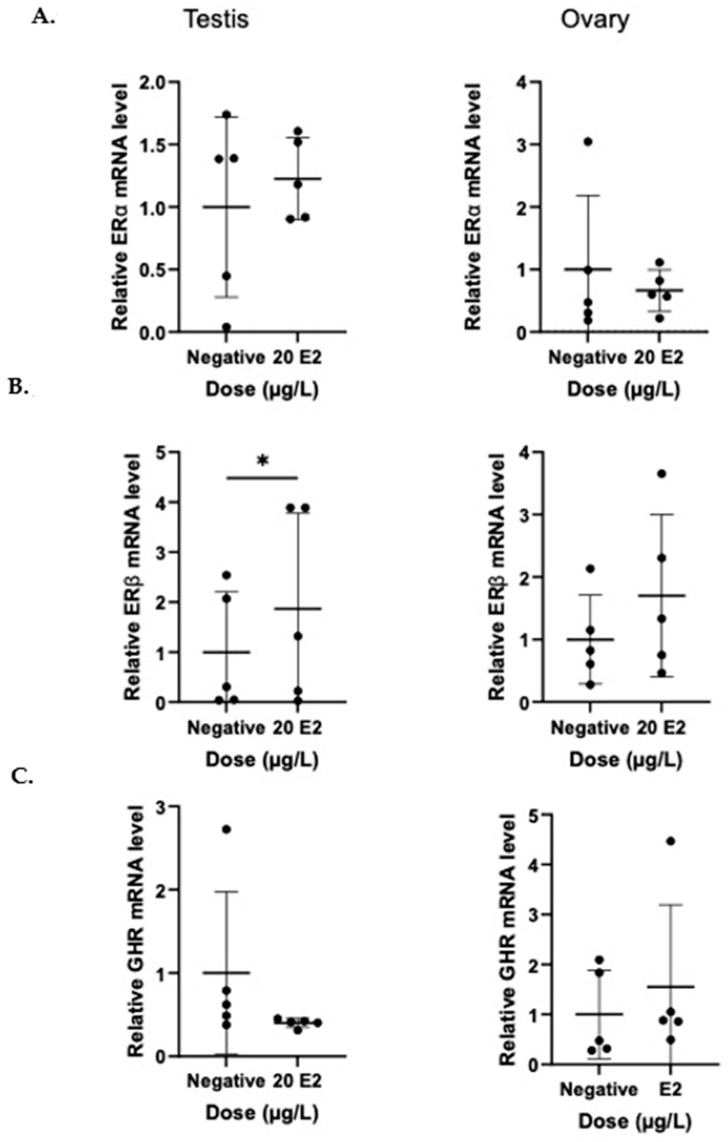
In vitro treatment of 20 μg/L E2 causes increased mRNA gene expression of ERβ in tilapia (*Oreochromis mossambicus*) testes, but not ovaries. Gonadal tissue was cultured and treated with 20 μg/L E2 for 24 h at 25 °C and mRNA levels of (**A**) ERα, (**B**) ERβ, and (**C**) GRH were determined by quantitative real-time PCR (qPCR). Expression is presented relative to the internal control gene (β-actin), with respect to control (mean ± SD; * denotes statistical significance with *t*-test *p* < 0.05, n = 5).

After a 24 h treatment period, none of the BP-3 doses induced significant changes in the gene expression of ERα, ERβ, or GHR in male or female gonads (*p* > 0.05) (Figure 1). In vitro treatment for 24 h with a solution of 20 µg/L E2 caused a significant increase in ERβ mRNA expression in male testes tissue from tilapia compared to β-actin expression (*p* < 0.05) (Figure 2). In vitro treatment of tilapia ovarian tissue of 20 µg/L of E2 showed a trend towards increased ERβ expression, but this trend was not statistically significant (*p* = 0.054) (Figure 2). The physiological dose of E2 had no effect on the gene expression of Erα or GRH in either male or female gonadal tissue compared to β-actin expression over this 24 h period. While BP-3 did not cause any significant effects on gene expression after acute treatment, this is the first time autoregulation of ER expression has been shown in tilapia gonads after acute in vitro treatment.

## 4. Discussion

The goal of this study was to evaluate BP-3 as an endocrine disruptor in teleost fish. Specifically, we were interested in its ability to regulate the mRNA expression of reproductively important steroid hormones in the gonadal tissue. The reproductive physiology of Mozambique tilapia is similar to many other teleost species, making it a valuable model species for studying endocrine-disrupting chemicals in fish [31,32,33]. The present results indicate that the currently described environmental levels of BP-3 in aquatic ecosystems may not be great enough to exert direct effects on the mRNA expression levels of ERs or GHRs in tilapia gonads.

The results from this study reveal that acute in vitro treatment of BP-3 at environmentally relevant doses (up to 100 µg/L) does not cause any significant changes in the gene expression of reproductively important ERs and GHRs in tilapia gonads. While a handful of in vivo studies using very high doses (up to 1000 µg/L [41]) have shown that BP-3 can cause phenotypic endocrine-disrupting effects, such as a female-biased population [25], decreasing egg viability and hatching [29], and increasing plasma vitellogenin in males [29], the mechanism of these effects remains unclear. The results from this study suggest that within aquatic systems contaminated with BP-3, there may not be direct effects on the gene expression of reproductive ERs or GHRs in teleost fish. This lack of significant effect of BP-3 on gene regulation in vitro after acute treatment may indicate that the endocrine-disrupting effects of this compound on teleost fish may not be as robust as originally postulated. This is important, as the impact of organic UV filters on aquatic ecosystems has become an important area of research, particularly as many countries have begun to impose sunscreen bans as a result of the perceived dangers of these compounds on coral and wildlife.

These results may not correlate with the mechanism exerted on tissue with the relatively high dosages that have been used in in vivo studies. These findings also do not rule out the possibility that BP-3, and similar organic UV filters, can cause endocrine-disrupting effects on aquatic life, as these experiments were conducted under limited conditions. Specifically, the cell culture was not viable for an extended period of time; therefore, our study was limited to an exposure time of 24 h. It is possible that this time period was not long enough for BP-3 to exert an effect.

Directly treating gonadal tissue with the parent compound, BP-3, rather than an active metabolite, may not accurately simulate in vivo conditions. Studies have found that BP-3 is extensively metabolized in vivo to more than twenty metabolites, including BP-1, BP-2, and, most notably, 4-hydroxybenzophenone (4-OH-BP) [42]. While it is known that 4-OH-BP is a major metabolite of BP-3 and can bioaccumulate in animals [43], its toxic potential has not been studied in depth. It has been shown, however, that 4-OH-BP exerts more estrogenic effects in human breast cancer cell lines than BP-3 [44]. Further in vitro study designs may be warranted to explore the physiological effects of the metabolites of BP-3.

The mechanism by which BP-3 exerts any estrogenic effects in vivo also remains unclear. The findings from this study suggest that it is not via BP-3 acting directly on ERs in the gonadal tissue. It is therefore possible that BP-3 inhibits androgenic effects, rather than primarily inducing estrogenic effects, as some in vivo studies suggest [45]. EHMC (2-ethyl-hexyl-4-trimethoxycinnamate), an organic UV filter that is similar to BP-3, has been shown to upregulate aromatase (cyp19a) in fish, which is the enzyme that converts testosterone to estradiol [46,47]. This increase in cyp19a could contribute to the potential estrogenic effects of organic UV filters, as well as decreasing androgenic effects. Future studies can utilize the in vitro methodology that is successfully implemented in this study using different target genes to examine the potential anti-androgenic effects of BP-3.

This study describes the autoregulation of estrogen receptors in the gonadal tissue of tilapia. Estrogen in male tilapia upregulates ERβ expression, thereby amplifying the effect of estrogen. It is likely that this is also the case in female tilapia, and it is possible that we failed to detect this effect because female fish can be sensitized to E2, prior to excision. Estrogen is an important steroid hormone that helps mediate reproductive cycles and gonadal development in teleost fish [30,48]. These reproductive cycles are tightly regulated through complex feedback loops involving the interactions of various hormones, including GTH (gonadotropin), GnRH (GTH-releasing hormone), THs (thyroid hormones), androgens, and estrogens [49,50]. While these hormones interact with each other to elicit synchronous effects, many of them have been shown to have direct acute effects on tissues in various fish species in vitro. Estrogen, in particular, has species-specific effects on gene regulation in the gonadal tissue of fish [48].

The results from this study highlight the need for more in vitro study designs to begin to uncover the estrogenic mechanism of action of organic UV filters on aquatic species observed in in vivo designs. BP-3 can bioaccumulate in the brain of crucian carp [51] and can regulate GnRH [52,53]. Therefore, culturing different tissues in the HPG axis using this methodology, particularly the pituitary gland, could potentially reveal if BP-3 is interacting with other reproductive targets that could cause a cascading estrogenic effect.

## 5. Conclusions

This study provides initial evidence that environmentally relevant doses of BP-3 do not cause changes in gene expression in tilapia gonads after acute treatment. Although there was no significant effect of BP-3, these negative data are still important. Governments regulating sunscreen ingredients and tourist management are relying on previous results obtained from treatment with extremely high doses that are not observed in natural habitats. These results provide preliminary data that the current levels of organic UV filters in natural aquatic environments may not affect teleost fish reproduction as negatively as originally proposed. This highlights the need for future research on the impact of these filters on aquatic organisms.

Future studies should focus on determining the lowest effective concentration (LOEC) of BP-3 to induce gene regulation in gonadal tissue in vitro, both for acute and long-term treatments. It is important to note that this study design included only one time point, 24 h, and the next steps should include shorter and longer timepoints. It would also be informative for future in vivo study designs to measure the amount of organic UV filter accumulated in various fish tissues after long term exposure to allow for comparison between significant effects and accumulation levels.

This work examined the effects of one potential endocrine-disrupting chemical that is currently in aquatic ecosystems worldwide. There are a myriad of other anthropogenic pollutants contaminating Earth’s bodies of water. For this reason, it is important to continuously study these compounds and the potential harmful effects they could have on aquatic organisms. To determine which compounds are safe for aquatic ecosystems and which are not, it is first important to understand their mechanisms of action using in vitro study designs. The in vitro methodology in this study has many potential uses for future work on many other environmental pollutants.

## Data Availability

All data is contained within the article. Additionally, raw data numbers can be made available on request by the corresponding author.

## Abbreviations

The following abbreviations are used in this manuscript:

BP-3	Hydroxy-4-methoxybenzophenone
4MBC	4-methylbenzylidene camphor
GHRs	Growth hormone receptors
ERs	Estrogen receptors
MS-222	Methanesulfonate
LOEC	Lowest effective concentration
IACUC	Institutional Animal Care and Use Committee
PBS	Phosphate-buffered saline
L15	Leibovitz’s 15 media
FBS	Fetal bovine serum
E2	Estrogen
4-OH-BP	4-hydroxybenzophenone
EHMC	2-ethyl-hexyl-4-trimethoxycinnamate
GnRH	Gonadotropin-releasing hormone
TH	Thyroid hormone
GTH	Gonadotropic hormone
HPG	Hypothalamic–Pituitary–Gonadal
